# Impact of Antigen Presentation Mechanisms on Immune Response in Autoimmune Hepatitis

**DOI:** 10.3389/fimmu.2021.814155

**Published:** 2022-01-18

**Authors:** Rossella Fasano, Eleonora Malerba, Marcella Prete, Antonio Giovanni Solimando, Alessio Buonavoglia, Nicola Silvestris, Patrizia Leone, Vito Racanelli

**Affiliations:** ^1^ Department of Biomedical Sciences and Human Oncology, “Aldo Moro” University of Bari Medical School, Bari, Italy; ^2^ Medical Oncology Unit, Istituto di Ricovero e Cura a Carattere Scientifico (IRCCS) Istituto Tumori Giovanni Paolo II, Bari, Italy

**Keywords:** autoimmune hepatitis, major histocompatibility complex class II, major histocompatibility complex class I, antigen processing and presentation, liver

## Abstract

The liver is a very tolerogenic organ. It is continually exposed to a multitude of antigens and is able to promote an effective immune response against pathogens and simultaneously immune tolerance against self-antigens. In spite of strong peripheral and central tolerogenic mechanisms, loss of tolerance can occur in autoimmune liver diseases, such as autoimmune hepatitis (AIH) through a combination of genetic predisposition, environmental factors, and an imbalance in immunological regulatory mechanisms. The liver hosts several types of conventional resident antigen presenting cells (APCs) such as dendritic cells, B cells and macrophages (Kupffer cells), and unconventional APCs including liver sinusoidal endothelial cells, hepatic stellate cells and hepatocytes. By standard (direct presentation and cross-presentation) and alternative mechanisms (cross-dressing and MHC class II-dressing), liver APCs presents self-antigen to naive T cells in the presence of costimulation leading to an altered immune response that results in liver injury and inflammation. Additionally, the transport of antigens and antigen:MHC complexes by trogocytosis and extracellular vesicles between different cells in the liver contributes to enhance antigen presentation and amplify autoimmune response. Here, we focus on the impact of antigen presentation on the immune response in the liver and on the functional role of the immune cells in the induction of liver inflammation. A better understanding of these key pathogenic aspects could facilitate the establishment of novel therapeutic strategies in AIH.

## Introduction

The liver is an important immunological organ with the unique capacity to mount effective immune responses against hepatotropic pathogens on one hand and maintain a local and systemic immune tolerance to self and foreign antigens on the other (1). The liver’s immune function is strongly influenced by its exclusive anatomy and by its cell composition (1). The liver hosts both conventional such as dendritic cells (DCs), B cells and macrophages (Kupffer cells, KCs), and unconventional antigen presenting cells (APCs) including liver sinusoidal endothelial cells (LSECs), hepatic stellate cells (HSCs), and hepatocytes (HCs) which uptake antigens, process and present them to CD4^+^ and CD8^+^ T cells by canonical and noncanonical mechanisms resulting in initiation and amplification of immune responses or in induction of immune tolerance (1, 2). When this equilibrium is disrupted, the immune tolerance is lost developing autoimmune liver diseases such as autoimmune hepatitis (AIH) (1, 2). Moreover, intercellular transfer of the peptide-major histocompatibility complexes (MHCs) *via* trogocytosis and extracellular vesicles (EVs) can confer to any cell APC features, albeit with different outcomes. Upon self-antigen presentation by liver APCs, the activation of a variety of immune cells such as Th0-, Th1- and Th2-CD4^+^ T cells, Th17 cells, cytotoxic CD8^+^ T cells, regulatory T cells (Treg), natural killer (NK) cells and B cells, along with the release of cytokines including interferon (IFN)-γ, transforming growth factor-β (TGF-β), interleukin (IL)-10, IL-21, IL-2 and autoantibodies results in autoimmune attack of liver in AIH.

The pathogenesis of AIH is complex and so far not fully understood. Growing evidence suggests that molecular mimicry and enhanced autoantigen presentation contribute to trigger autoimmune response resulting in the activation of autoreactive lymphocytes.

Current treatments for AIH focus on non-specific immunosuppressive drugs and do not rely on the immune pathology underlying the autoimmune response. A better understanding of the impact of alternative antigen presentation mechanisms on the immune response in the liver and of the functional role of the immune cells in the induction of liver inflammation could facilitate the establishment of novel therapeutic strategies in AIH (3). The development of antigen-specific immunotherapy aiming to manipulate antigen presentation and reprogram APCs towards a tolerant phenotype may represent effective therapeutic strategies for treatment of refractory AIH.

## Autoimmune Hepatitis

AIH is a rare acute or chronic inflammatory liver disease clinically presenting with high levels of circulating autoantibodies, hypergammaglobulinemia, elevated serum aminotransferase levels and interface hepatitis on histological examination with a lymphoplasmacytic infiltrate (4). According to the autoantibodies detected at diagnosis can be identified two subsets of AIH: type 1 autoimmune hepatitis (AIH-1), defined by the presence of anti-nuclear antibody (ANA) and/or anti-smooth muscle antibody (SMA), and type 2 autoimmune hepatitis (AIH-2) associated with positivity for anti-liver/kidney-microsomal-antibody-type-1 (anti-LKM-1) or anti-liver-cytosol-type-1 (anti-LC1) autoantibodies (3, 5). Clinical presentations are variable; patients may be asymptomatic, chronically ill, or present with acute or fulminant liver failure (3). Concurrent autoimmune diseases are frequently observed (6). Incidence and prevalence vary according to age, gender, ethnicity, and geographical region (7). Based on European studies, the annual incidence ranges from 0.9 to 2.0 cases per 100,000 persons and the annual prevalence ranges from 11 to 25 cases per 100,000 individuals, depending on the geographical location (8). AIH occurs globally in all ethnicities and affects children and adults of all ages. AIH-1 displays a bimodal age pattern at presentation, with one peak during childhood or adolescent and the other in the adulthood around the age of 40 years. AIH-2 is typical of pediatric ages and is rare in adults (9). As many autoimmune diseases, AIH has a female predominance (3, 7).

**Figure 1:**
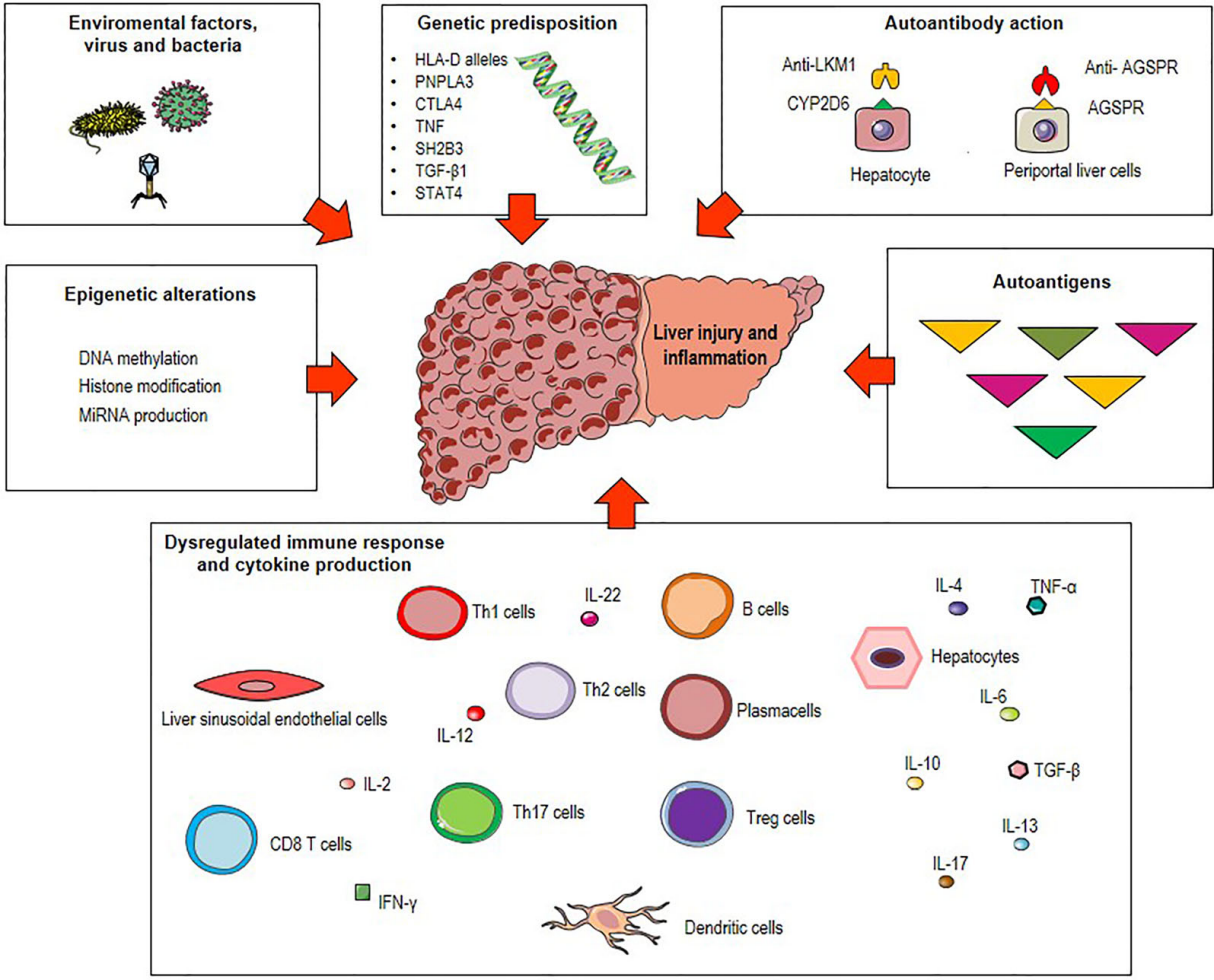
Possible triggers of autoimmune hepatitis (AIH). Environmental factors (microbial products, drugs metabolites), epigenetic alterations (DNA methylation, histone modification and miRNA production) and genetic predisposition including polymorphisms of the human leukocyte antigen (HLA) genes (HLA class II DRB1 alleles), the Src homology 2-B adaptor protein 3 gene (SH2B3), the patatin-like phospholipase domain-containing protein 3 (PNPLA3), the cytotoxic T lymphocyte-associated antigen 4 (CTLA4), the tumor necrosis factor (TNF), the transforming growth factor-β1 (TGF-β1) and the signal transducer and activator of transcription (STAT) 4 are associated with great risk of developing AIH. Combination of these factors with pathogenetic mechanisms such as molecular mimicry, autoantibody production and dysregulated immune responses against liver autoantigens lead to immune tolerance breakdown and consequent liver injury and inflammation.

The precise etiology of AIH remains unknown. Along with genetic, epigenetic and environmental factors, pathogenic mechanisms such as molecular mimicry, altered antigen presentation, and dysregulated immune responses against liver autoantigens can lead to immune tolerance breakdown (3, 10–22) (**Figure 1**). Existing therapies for AIH are based on non-specific immunosuppressive drugs and do not consider key immunopathological aspects underlying the initiation and perpetuation of the autoimmune response. Among these, the presentation of self-antigenic peptides from liver APCs to T cells deserves to be explored. Therefore, increasing knowledge about mechanisms of antigen presentation could help to design novel therapeutic strategies to reestablish immunological tolerance and to treat unstable or refractory AIH.

## Initiation of the Autoimmune Response

The mechanisms underlying AIH pathogenesis are not fully elucidated, although there is growing evidence that molecular mimicry and enhanced autoantigen presentation are involved in the induction of the autoimmune response resulting in the activation of autoreactive lymphocytes.

### Molecular Mimicry

“Molecular mimicry” is a process where a foreign antigen shares sequence or structural similarities with self-antigens leading to autoimmunity. In this case, the immune system can be confused by the foreign antigen which induces an immune response towards similar self-antigens (23). Viral infections have been reported to be risk factors for AIH and several mouse models for AIH demonstrated that molecular mimicry is a prerequisite for breaking cell tolerance in the liver (24–26). During chronic hepatitis B virus (HBV) and hepatitis C virus (HCV) infections, approximately 50% of patients develop autoantibodies such as ANA and SMA as a consequence of cross-reactive immune reactions between host smooth muscle/nuclear components and HCV antigens (27, 28). Moreover, 10% of chronic HCV patients reported to be positive for anti-LKM-1 autoantibodies. This positivity is due to a high amino acid sequence homology between HCV polyprotein and cytochrome P450 2D6 (CYP2D6), the target of anti-LKM-1 antibodies (29, 30).

### Antigen Presentation

**Figure 2:**
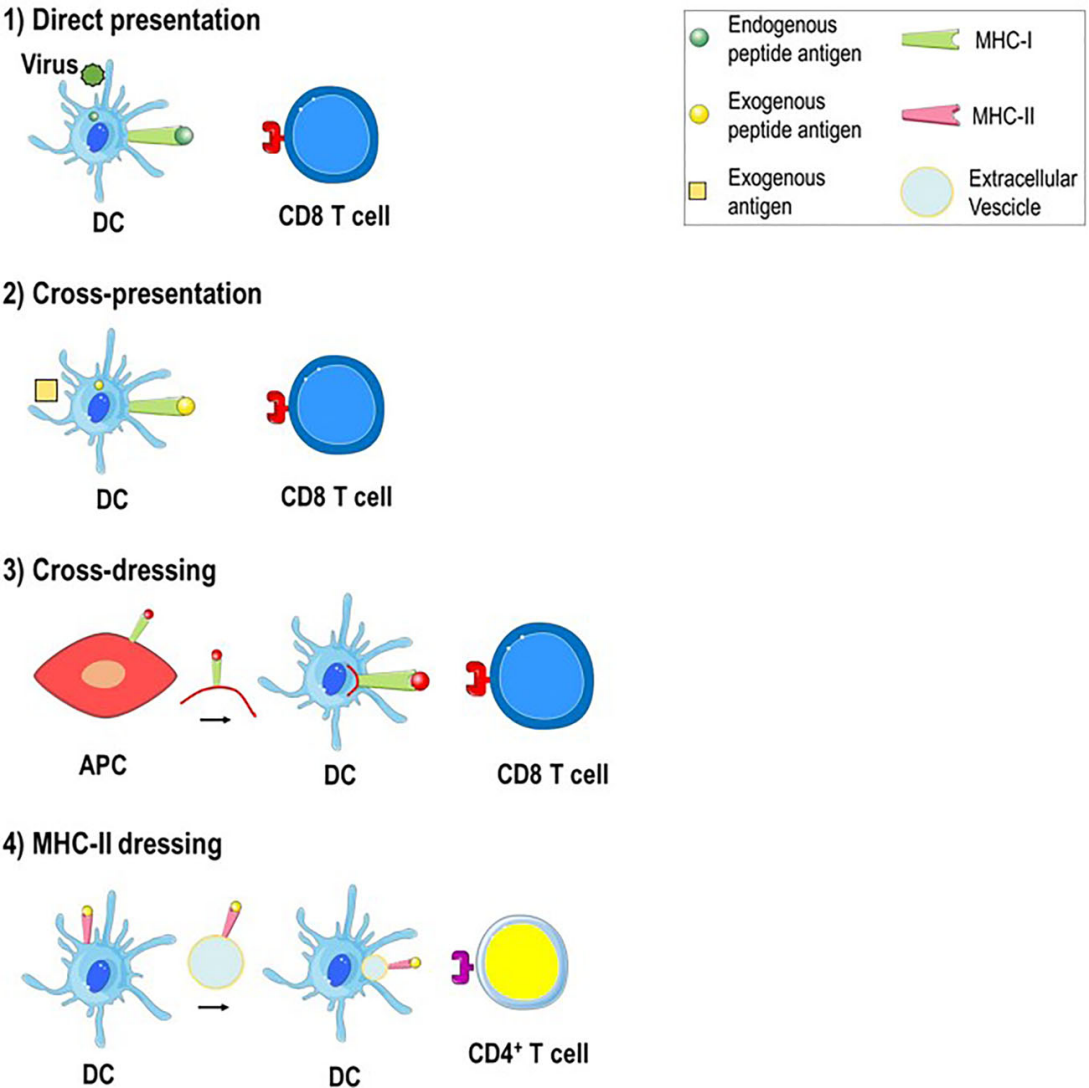
Mechanisms of antigen presentation in the liver. 1) Direct presentation. Within virus-infected DC, the endogenous antigen is broken down into peptides that bind to endogenous MHC class I molecules and are presented to antigen-specific CD8^+^ T cells. 2) Cross-presentation. An exogenous antigen, such as a virus that do not infect DC, is engulfed by DC, processed and derived peptide antigens are presented to CD8^+^ T cells through MHC class I molecules. 3) Cross-dressing. The peptide-MHC class I complex is transferred from APC to DC by trogocytosis or extracellular vesicles. 4) MHC-II dressing. The peptide-MHC class II complex is transferred (through trogocytosis/extracellular vesicles) from DC to neighboring DC which can use it to activate CD4^+^ T cells.

Professional APCs are able to process intracellular and extracellular pathogens through different pathways. In the MHC class I antigen presentation pathway, endogenous antigens are digested by the proteasome (an intracellular protease complex) and presented by MHC class I molecules to CD8^+^ T cells to induce a cell-killing response. In the MHC class II antigen presentation pathway, exogenous antigens are engulfed by APCs such as DCs, digested in the phagolysosome and presented by MHC class II molecules to CD4^+^ T cells to stimulate a helper response (31, 32). Antigen presentation can take place in the following direct and indirect ways: 1) direct presentation, 2) cross-presentation, 3) cross-dressing and 4) MHC class II dressing (**Figure 2**). 1) Direct presentation occurs when the APC such as DC is infected by a virus and the endogenous viral proteins are broken down in the cytosol into peptides that, through the MHC class I antigen presentation pathway, bind to endogenous MHC class I molecules and are presented to naive CD8^+^ T cells to generate virus-specific cytotoxic CD8^+^ T cells. 2) Cross-presentation arises when exogenous antigens, such as viruses do not infect professional APC, are engulfed by DC, escape from phagosomes to enter the cytosol and gain access to MHC class I molecules to stimulate CD8^+^ T cells (33). 3) Cross-dressing takes place by intercellular transfer of the peptide-MHC class I complex from an APC or tumor cell to a DC through trogocytosis, exosomes or tunneling nanotubes. This process results in activation of cytotoxic CD8^+^ T cells and is defined as “DC cross-dressing” (34). 4) MHC class II dressing occurs when the exogenous peptide-MHC class II complex is transferred (through trogocytosis/exosomes) from DCs to nearby cells such as DCs, CD4^+^ T cells, or type 2 innate immune cells. This intercellular transfer involves two live cells, requires cell-to-cell contact and result in CD4^+^ T cell activation (34–36). All the antigen presentation mechanisms mentioned above are involved in the pathogenesis of AIH.

#### Direct Presentation and Cross-Presentation in AIH

In normal conditions, the liver displays a tolerogenic capacity resulting from a combined effort of immune cells, parenchyma cells, epithelial and endothelial cells and microenvironment. Because of the high local concentrations of the immunosuppressive cytokine IL-10, the majority of liver APCs, including DCs, LSECs, KCs and HCs, have low levels of MHC class II and the co-stimulatory molecules CD80/CD86, thus exhibit an immature phenotype which induces immune tolerance (3). The priming of hepatic CD4^+^ T cell leads to the induction of tolerogenic Foxp3^+^ and Foxp3^+^ IL-10^+^ CD4^+^ Tregs which further release IL-10 augmenting the immune suppressive milieu (37–40). In addition, hepatic CD8^+^ T cell priming induces deletion of premature and nonantigen-specific CD8^+^ T cells (2). The effect of T cell priming, however, depends also on the overall antigen load; when the antigen density is low, T cells are activated and an effector response is initiated, whereas when the antigen load is high, T cell priming results in T cell anergy and exhaustion due to the great expression on the surface of the programmed cell death protein 1 (PD-1) (2). Moreover, the site of primary T cell activation influences the outcome of intrahepatic CD8^+^ T cell responses. Using a transgenic mouse model in which antigen is expressed within both liver and lymph nodes, Bowen et al. demonstrated that CD8^+^ T cells primed within lymph nodes generated an effective intrahepatic antigen-specific immune response, whereas CD8^+^ T cells activated within the tolerogenic environment of the liver induced a defective cytotoxic immune response and showed a short half-life (41). During autoimmune diseases such as AIH, a breakdown in hepatic tolerance takes place (42). The trigger underlying the switch towards an overwhelming immune response against autoantigens is still unknown. From an immunological view, the autoantigen presentation by conventional and unconventional liver APCs, the unsuccessful control of autoreactive T cells and the production of autoantibodies play a crucial role (43, 44). Dysregulated Treg cells which are also susceptible to Fas ligand-mediated apoptosis can be observed within the liver of AIH patients (45–48). Moreover, during AIH, beyond their function as autoantibody-secreting cells, B cells can act as potent APCs activating autoreactive CD4^+^ T cells through the MHC class II antigen presentation pathway (49, 50). This B-T cell interaction generates a vicious cycle, a positive feedback loop in which autoreactive B cells as APC activate CD4^+^ T cells which in turn stimulate B cells leading to their terminal differentiation in immunoglobulin-producing plasma cells and to amplification of autoimmunity (51). The importance of B cells as APC in AIH pathogenesis is suggested by B cell depletion with anti-CD20 antibody in mouse models. Anti-CD20 antibody treatment significantly reduced expression of MHC class II and CD80 on B cells with consequent diminished capacity of autoantigen presentation to T cells and reduction of T cell activation and proliferation (52). Moreover, a single dose of anti-CD20 antibody was able to induce remission of liver inflammation, reduced hepatocytes lysis and consequent lowered autoantigen release (52).

#### Cross-Dressing and MHC-II Dressing in AIH

The context in which the MHC complexes are expressed impacts the outcome of T cell priming. During homeostasis, HCs exhibit only MHC class I molecules on their surface. During infectious and autoimmune liver diseases, such as AIH, the inflamed microenvironment induces the aberrant expression of MHC class II molecules by HCs, which acquire the capacity to perform antigen-presenting cell functions (53) and to elicit Th1 or Th2 effector responses (54). However, the acquired capacity of HCs to present antigen on MHC class II molecules is not sufficient to cause AIH (54). Further investigations are required to clarify the increased risk for AIH conferred by the expression of certain human leukocyte antigen (HLA)-DR and HLA-DQ loci (14, 15).

**Figure 3:**
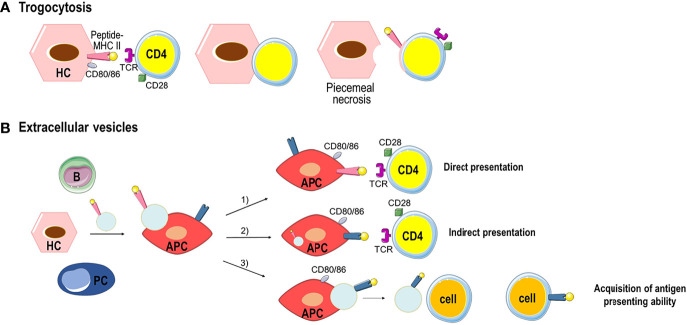
Examples of peptide-MHC complex transfer by trogocytosis or extracellular vesicles. **(A)** Trogocytosis between MHC class II-expressing HCs and CD4^+^ T cells resulting in the transfer of the membrane fragment with the peptide-MHC class II complex to CD4^+^ T cells. HCs that lose part of their membrane go to death (piecemeal necrosis). **(B)** All parenchymal and nonparenchymal cells produces extracellular vesicles. Extracellular vesicles bearing peptide-MHC class II complexes are taken up by APC triggering three separate immune responses: 1) direct presentation of antigen and priming of CD4^+^ T cells. 2) the peptide-MHC class II complex is processed by the APC, and the antigen peptide is bound to the MHC class II molecules of APC to elicit indirect antigen presentation and CD4^+^ T cell priming. 3) after the processing the antigen peptide is bound to the MHC class II molecules of APC and the new peptide-MHC class II complex is transferred through extracellular vesicles to other cells which acquire antigen presenting ability.

During AIH, trogocytosis can occur between MHC class II-expressing HCs and CD4^+^ T cells in a TCR dependent manner resulting in peptide-MHC class II transfer onto CD4^+^ T cells and dying HCs that lose part of the hepatocyte membrane (55, 56) **(** **Figure 3A** **).** This process named piecemeal necrosis is characterized by necrosis of periportal HCs with inflammation extending from the portal tract into the periportal zone (57). The transfer of peptide-MHC class II complexes along with co-stimulatory molecules, such as CD80/CD86 to CD4^+^ T cell, can enable T cell to act as APC, inducing naïve T cell activation and amplification of the effector T cell response. In the absence of co-stimulatory signals, tolerance is established leading to T cell apoptosis and hyporesponsiveness (43).

Extracellular vesicles (EVs) are an alternative way of transferring cellular content from one cell to another (58). EVs are lipid bilayer vesicles originating from different cell types. Based on their size, they can be distinguish in exosomes (50–150 nm), microvesicles (100–1000 nm) and apoptotic bodies (500–2000 nm) (59). EVs/exosomes can be equipped with a multitude of molecules and soluble factors with immunomodulatory functions. In the liver, after binding to target cells such as APCs, EVs/exosomes carrying peptide-MHC class II complexes are taken up by endocytosis and can induce three different immune responses (**Figure 3B**): 1) the peptide-MHC class II complex is directly presented by the APC to CD4^+^ T cell eliciting T cell priming in an immunological synapse that involves co-stimulatory molecules; 2) the peptide-MHC class II complex is processed by the APC, and the antigen peptide is bound to the MHC class II molecules of APC, and presented to CD4^+^ T cell to elicit indirect antigen presentation and T cell priming; 3) after the processing of the peptide-MHC class II complex, the antigen peptide is bound to the MHC class II molecules of APC and the new peptide-MHC class II complex is released by the APC by EVs that could transfer the complex to remote cells transferring antigen presenting ability (43).

Beside peptide-MHC class II complexes, liver EVs/exosomes can also carry Fas ligand (FasL/CD95L) that communicates cell death signals when it binds to its receptor, Fas/CD95, integrins, the asialoglycoprotein receptor usually expressed by HCs, as well as soluble factors like TGF-β and TNF-α (60–62).

EVs and trogocytosis make possible also the transfer of the MHC class I molecules. During viral infections, intercellular transfer of MHC class I molecules has been observed in the liver from the HSCs to LSECs. Such transfer supports LSEC cross-presentation of antigens to CD8 T cells reinforcing the cytotoxic T cell response (63). It is yet unclear whether this mechanism occurs also during liver autoimmune disease such as AIH sustaining the priming of autoreactive CD8 T cells (43).

### Immune Cells Activated Upon Self-Antigen Presentation

During AIH, the processing and presentation of self-antigens by liver APC initiates the immune response through the activation of both naïve CD4^+^ T helper (Th0) cells and CD8^+^ effector T cells in the presence of co-stimulatory molecules as CD80/86 on APC and CD28 on T cells (64). Upon activation, CD4^+^ Th0 cells can differentiate into various T helper cell populations according to the cytokines in the microenvironment and the nature of the antigen.

**Figure 4:**
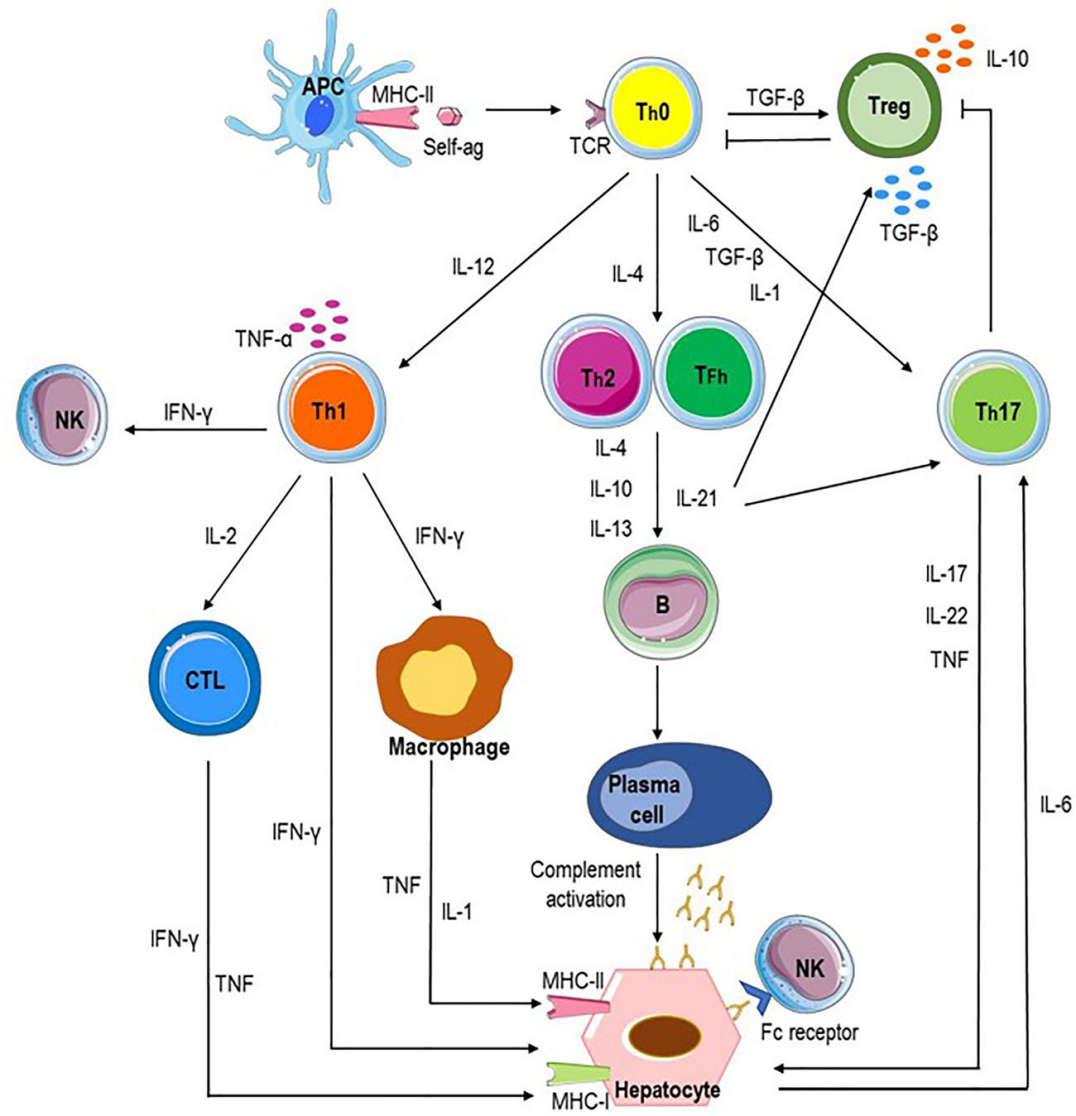
Potential pathways of autoimmune attack of hepatocytes in AIH. Autoimmune-mediated liver damage in AIH is likely induced by an immune response to liver autoantigens. Antigen-presenting cells (APC) present autoantigenic peptide to naive CD4^+^ T helper cells (Th0), which release proinflammatory cytokines (IL-1, IL-4, IL-12, IL-6, and transforming growth factor beta, TGF-β) resulting in the differentiation of regulatory T cells (Treg), Th1, Th2, and Th17 cells. Each cell subset releases cytokines which lead to a cascade of events culminating with the autoimmune attack of hepatocytes. Liver cell destruction could derive from the activation of cytotoxic T lymphocytes (CTL) through cytokines released by Th1 and activated macrophages; Th2 cells secrete IL-4, IL-10, and IL-13 which trigger B cell maturation into plasma cells which produce autoantibodies and induce antibody-dependent cellular cytotoxicity and complement activation. Th17 cells release proinflammatory cytokines such as IL-17, IL-22, and tumor necrosis factor alpha (TNF-α), inhibit Treg cells and stimulates hepatocytes to secrete IL-6 which further amplifies Th17 cell activation. Tfh (T follicular helper) cells participate to activation and differentiation of B cells in antibody-secreting plasma cells and release IL-21, a regulator of Treg cell differentiation and Th17 cell expansion.

A microenvironment with high levels of transforming growth factor-β (TGF-β) promotes differentiation of Th0 cells into Treg cells; the predominance of IL-12 supports the differentiation of Th0 lymphocytes into Th1 cells, whereas an IL-4-rich milieu favors the differentiation into Th2 cells. Furthermore, TGF-β, IL-1, and IL-6 promote differentiation into T helper 17 (Th17) cells (3). Each cell subset releases cytokines which lead to a cascade of events culminating with the autoimmune attack of hepatocytes (**Figure 4**).

Treg cells are key players in autoimmune diseases including AIH. Functional impairment and altered frequency of Treg cells in AIH patients cause breakdown of self-tolerance mechanisms (46) with consequent beginning and progression of autoimmune liver injury (44, 65). In patients with AIH, the frequency of circulating Treg cells and their ability to suppress T cell proliferation are lower than in healthy subjects, especially at diagnosis and during relapses than during drug-induced remission (66–68). Treg cells are characterized by the expression of markers associated with the acquisition of regulatory properties, including Foxp3, CTLA4 and the glucocorticoid-induced TNF receptor, CD62 ligand. Importantly, they express little or no IL−7 receptor (CD127). They act by direct contact with the target cells and by secreting inhibitory cytokines, such as IL−10 and TGF-β (69). Moreover, the expression of the ectoenzyme CD39 in concert with CD73/ecto-5’-nucleotidase distinguishes on Treg cells and the adenosine A2A receptor on activated effector T cells creates immunosuppressive loops (70–72). It has been shown that CD39^+^ Treg cells are reduced in AIH patients, fail to adequately hydrolyze proinflammatory nucleotides, and do not effectively suppress IL-17 production by effector CD4^+^ T cells (73). In addition, CD39^+^ Treg cells are plastic and unstable in the presence of proinflammatory stimuli switching from Treg to effector cells, and decreasing further Treg frequency (73).

Th1 cell differentiation results in the secretion of IL-2, INF-γ and TNF-α and in the activation of cytotoxic CD8^+^ T lymphocytes (CTLs) which, after interaction with antigens presented by the MHC class I-antigenic peptide complexes on hepatocytes, attack hepatocytes causing liver damage (10, 74, 75). INF-γ is a crucial mediator of tissue damage since it not only stimulates CD8^+^ T cells, increases the expression of MHC class I, and promotes aberrant expression of MHC class II molecules on hepatocytes, making them able to present antigens on their surface, thus enhancing T cell activation and perpetuating liver damage (44). Moreover, INF-γ activates monocytes/macrophages, which in turn release IL-1 and TNF-α (76) and enhances NK cell activity (77). AIH-2 patients are characterized by higher percentage of peripheral blood CD8^+^ T cells producing IFN-γ at diagnosis than during effective immunosuppressive treatment (78) and by liver inflammatory infiltrate rich in IFN-γ-producing Th1 cells (79). AIH-2 patients have liver inflammatory infiltrates rich in Th1 cells producing TNF-α (10).

Th2 cells release IL-4, IL-10, and IL-13 which trigger B cell maturation into plasma cells secreting autoantibodies which can cause liver injury through antibody-dependent cellular cytotoxicity and complement activation (80). The presence of plasma cells in the damaged liver and of circulating autoantibodies are a key feature of AIH and are used as diagnostic and classification markers (81) (**Table 1**). Furthermore, the expression on the surface of hepatocytes of CYP2D6, the target of anti-LKM1 autoantibodies exposes them to direct humoral immune assault (82).

**Table 1:** Autoantibodies in AIH.

AUTOANTIBODY	TARGET ANTIGEN	DISEASE	PERCENTAGE
ANA	Chromatin, histones, centromeres, (ds) and (ss) DNA, cyclin A, ribonucleoprotein	AIH-1	50-70%
SMA	F-actin, actin, tubulin, intermediate filaments	AIH-1	50%
Anti-LKM-1	Cythocrome P4502D6	AIH-2	85%
Anti-LKM-3	Uridine-diphosphate-glucuronosyl-transferase	AIH-2	rare
Anti-SLA/LP	O-phosphoseryl-tRNA: selenocysteine-tRNA synthase (SepSecS)	AIH-1	10-20%
		AIH-2	
p-ANCA	Various cytoplasmatic antigens	AIH-1	36-50%
Anti-LC-1	Forminotransferase cyclodeaminase	AIH-2	30%
Anti-ASGP-R	Asialoglycoprotein receptor	AIH-1	24-82%
		AIH-2	

AIH, autoimmune hepatitis; ANA, antinuclear antibody; SMA, anti-smooth muscle antibody; Anti- LKM-1, anti-liver/kidney-microsomal-antibody-type-1; Anti-LKM-3, anti-liver/kidney-microsomal-antibody-type-3; Anti-SLA/LP, anti-soluble liver antigen/liver pancreas antigen; p-ANCA, perinuclear anti-neutrophil cytoplasmic antibody; anti-LC-1, anti-liver cytosolic antigen type 1; Anti-ASGP-R, anti-asialoglycoprotein receptor; ds, double strands; ss, single strand.

Th17 cells produce proinflammatory cytokines such as IL-17, IL-22, and TNF-α, support the autoimmunity process through inhibition of Treg and stimulates hepatocytes to secrete IL-6 which further amplifies Th17 cell activation (83, 84). Their number correlates with the severity of liver fibrosis (84).

New evidence suggests a role of Tfh (T follicular helper) cells in the AIH pathogenesis (85). Tfh cells are a subset of helper CD4^+^ T cells which, through the production of IL-21 and the expression of costimulatory molecules such as CD40 ligand, are able to induce the activation and differentiation of B cells in mature plasma cells secreting immunoglobulins. IL-21 is a regulator of Treg differentiation and function through the mTOR pathway, drives Th17 expansion (86) and promotes differentiation of Tfh cells by autocrine signaling (87). Dramatically high serum levels of IL-21 are found in severe AIH patients and correlate with disease activity (88, 89). Furthermore, Tfh cells generate crucial signals that regulate the affinity maturation of germinal center B cells and alterations in their numbers and functions are involved in the loss of self-tolerance (90).

γδ T cells in patients with AIH release high amount of IFN-γ and granzyme B contributing to liver damage (66), although further investigations are needed to clarify their role. Clonal analysis experiments revealed the presence of both alphabeta (αβ) and gammadelta (γδ) T cells in peripheral blood and liver biopsies from AIH patients. Specifically, the majority of clones obtained from the peripheral blood are CD4^+^ T cells bearing the αβ T cell receptor (αβ TCR), while the largest numbers of clones generated from liver biopsies of the same patients are CD4^-^CD8^-^ cells bearing the γδ TCR or CD8^+^ αβ T cells (91). γδ T-cell clones obtained from the peripheral blood and the portal areas of pediatric AIH patients show cytolytic activity against hepatocytes (91, 92).

## Treatment of AIH

Most AIH patients treated with current therapies show long-term complete response to treatment, but remain under lifelong immunosuppressive therapy. About 10–20% of patients do not respond to current therapy and display disease progression to cirrhosis and end-stage liver disease (81, 93). Moreover, current treatments as well as biological therapies such as anti-TNF (infliximab) and anti−CD20 (rituximab), although effective, are associated with serious side effects due to the specific drug or the broad immunosuppression (93–95).

Ongoing clinical trials are evaluating new therapeutic options aiming at targeting B lymphocytes or expanding regulatory T cells to restore immune tolerance. The AMBER study (ADCC Mediated B-Cell depletion and BAFF-R Blockade, NCT03217422, phase 2/3) is the first randomized double-blind, placebo-controlled trial that is assessing the safety and efficacy of the anti-BAFFR antibodies (Ianalumab, VAY736) in adults with AIH not responding to standard therapies (https://clinicaltrials.gov/ct2/show/NCT03217422). Further trials are evaluating the possible use of anti-BAFF drugs, such as belimumab (96), in refractory AIH patients with advanced fibrosis.

The expansion and therapeutic application of polyclonal Tregs is currently a treatment option for AIH-1. An ongoing uncontrolled, open label phase I/IIa clinical trial (NCT01988506) is investigating the employ of low-dose IL-2 therapy to enhance Treg functions in autoimmune and inflammatory diseases including AIH. Preliminary results in two AIH patients displayed Treg expansion and activation, without effector T cell activation, and with a good safety (97). *In vitro* experiments have shown the chance to generate antigen-specific Tregs from AIH-2 patients able to suppress cytotoxic activity by CD8^+^ T cells (98). Nevertheless, further investigations are required to maintain a stable and functional Treg phenotype in the inflammatory liver microenvironment.

Better knowledge of antigen presentation mechanisms in AIH may also open new therapeutic avenues. For example, antigen specific immunotherapy designed to manipulate antigen presentation and restore immunological self-tolerance to hepatic autoantigens leaving the rest of the immune system to function successfully are evaluating in mouse models (99) and are described in the section future directions.

## Future Directions

Future orientation in successful therapies in AIH and others autoimmune disorders will be the detection and isolation of autoreactive T cells, the manipulation of antigen presentation and the education of liver-resident APCs and effector T cells (43, 100). Flow cytometry and peptide-MHC complex tetramers have been the gold standard for detecting antigen-specific CD8^+^ and CD4^+^ T cells for more than two decades (101). In mouse models of AIH (Ad-hFTCD-infected NOD mice) (102) it has been demonstrated that engineered peptide-MHC complex-coated nanoparticles loaded with relevant AIH specific peptides activated and expanded cognate CD4^+^ type 1 regulatory (Tr1) T cells and regulatory B cells, and reduced liver inflammation, fibrosis, and alanine aminotransferase (ALT) levels without compromise immunity against viruses (vaccinia, influenza), intracellular bacteria (Listeria), or metastatic (liver) allogeneic tumors (102–104).

In addition, the delivery of autoantigen peptides to LSECs by nanoparticles can induce antigen-specific Tregs enable to control autoimmunity in mice (105). Furthermore, naturally produced and modified EVs equipped with a variety of molecules could be innovative and strong weapons for transport of drugs to the liver (58, 106), and for manipulating antigen presentation and reprogramming APCs towards a tolerant phenotype, educating immune cells such as T cells through the expression of PD-L1 or suppressive cytokines.

## Conclusions

A better understanding of the pathogenetic mechanisms underlined the autoimmune attack to the liver during AIH, with special attention to the antigen presentation processes that trigger the immune response in the liver could suggest new therapeutic strategies for the treatment of AIH patients. Cross-presentation, trogocytosis, cross dressing and EVs are tools which provide crucial regulatory mechanisms to enhance antigen presentation in the liver, to trigger tolerance or inflammation in AIH.

The design of nanoparticles coated with peptide-MHC complex presenting disease antigens and engineered EVs could facilitate regulation of immune cell activation in a disease-specific manner. Therefore, the detection of disease-related antigens and antibody-secreting B cells, the fine-tuning of the antigen specific immunotherapy to reinstate immunological self-tolerance could open new therapeutic strategies for treatment of AIH patients in order to specifically blocking liver auto-attack with few side effects.

## Author Contributions

Conceptualization, PL and VR. Data curation, RF, EM, AB, AS, and NS. Writing, RF, EM, and PL. Supervision, PL and VR. All of the authors reviewed the manuscript, approved the draft submission, and accept responsibility for all aspects of this study. All authors have read and agreed to the published version of the manuscript.

## Funding

This work was supported by the Italian Association for Cancer Research (AIRC) through an Investigator Grant no. 20441 to VR. The sponsors of this study are non-profit organizations that support science in general. They had no role in gathering, analyzing, or interpreting the data.

## Conflict of Interest

The authors declare that the research was conducted in the absence of any commercial or financial relationships that could be construed as a potential conflict of interest.

## Publisher’s Note

All claims expressed in this article are solely those of the authors and do not necessarily represent those of their affiliated organizations, or those of the publisher, the editors and the reviewers. Any product that may be evaluated in this article, or claim that may be made by its manufacturer, is not guaranteed or endorsed by the publisher.
